# Factors associated with poor outcomes in patients with maple syrup urine disease in a tertiary government hospital: A retrospective cohort study

**DOI:** 10.1002/jmd2.12458

**Published:** 2024-11-17

**Authors:** Christine Mae S. Avila, Mary Ann R. Abacan

**Affiliations:** ^1^ Division of Clinical and Metabolic Genetics, Department of Pediatrics, Philippine General Hospital University of the Philippines Manila Philippines; ^2^ Institute of Human Genetics, National Institutes of Health University of the Philippines Manila Philippines; ^3^ College of Medicine University of the Philippiines Manila Philippines

**Keywords:** maple syrup urine disease, newborn screening, Philippines

## Abstract

This study aims to determine the factors associated with mortality and neurodevelopmental morbidity in patients with Maple Syrup Urine Disease (MSUD) seen at a tertiary hospital in the Philippines during a 10‐year period. The medical records of patients diagnosed with MSUD seen at Philippine General Hospital (PGH) from 2010 to 2019 were reviewed. Socioeconomic, healthcare, and clinical factors were determined. The association of these factors with mortality and neurodevelopmental morbidity (developmental delay and seizures) was evaluated through statistical analysis. Seventy‐five records of MUSD cases were available for review. Fifty‐five percent of patients had developmental delay and 57% had seizures. Mortality rate was 25%. Age at collection of newborn screening (OR 1.29, 95% CI 1.04–1.60, *p* = 0.022) and the number of metabolic crisis in a year (OR 5.4, 95% CI 1.5–19.0, *p* = 0.008) were significantly associated with increased mortality. Male sex (OR 2.78, 95% CI 1.06–7.26, *p* = 0.037) and dietary non‐compliance (OR 2.56, 95% CI 1.48–4.42, *p* = 0.001) were associated with increased developmental delay. Age above 5 years (OR 6.5, 95% CI 1.15–36.57, *p* = 0.034) and nosocomial infections (OR 6.96, 95% CI 1.33–36.53, *p* = 0.022) were associated with occurrence of seizures. In conclusion, among our cohort of MSUD patients, the age at collection of newborn screening and the number of metabolic crises annually were associated with increased mortality. Male sex, dietary non‐compliance, and nosocomial infections were associated with increased neurodevelopmental morbidity.

**Synopsis**

Maple syrup urine disease (MSUD) is the most common inborn error of metabolism in the Philippines with a cumulative incidence of 1 in 55, 241 newborns in 2014. Despite early detection through the newborn screening program, mortality and neurodevelopmental morbidities are still observed. This study highlights socioeconomic, biochemical and healthcare‐associated factors that are potentially associated with hospital mortality, developmental delay or intellectual disability and occurence of seizures in our cohort of patients in one of the major island groups in the Philippines over a 10‐year period.

## 1 INTRODUCTION

Inborn errors of metabolism (IEMs) are rare disorders that are often neglected in most developing countries due to the burden of more common infectious, non‐communicable, and genetic disorders.^1^ However, these rare disorders need to be addressed since collectively around 2%–3% of the worldwide population suffers from IEMs. If the diagnosis is delayed or missed, the management of such patients becomes challenging and subsequently results in enormous financial and psychological burdens on the families and society.^1^, ^2^

In the Philippines, the most common inborn error of metabolism is maple syrup urine disease (MSUD). Its worldwide incidence is 1 in 185 000 live births.^3^ Before MSUD was included in the Newborn Screening (NBS) program, a total of only 47 patients were diagnosed in the country from 1992 to 2004.^4^, ^5^ When MSUD was added to the NBS in 2012, detection of the disease improved with the recent cumulative incidence documented to be 1 in 55 241 in 2014.^6^

Maple syrup urine disease is a condition wherein the body is unable to break down three branched‐chain amino acids (BCAA) which are leucine, isoleucine, and valine due to a deficiency in the branched‐chain alpha‐ketoacid dehydrogenase complex. Failure of breakdown of these proteins leads to the accumulation of toxic substances in the brain and other organs.^1^, ^7^, ^8^ The presentation of MSUD is very diverse and is comprised of different clinical phenotypes. The majority of the cases (75%) have the most severe classic form, which manifests during the neonatal period with extremely low enzymatic activity (0%–2%). The attenuated forms present later in life with higher enzymatic activity^3^, ^9^ but may still have severe manifestations.

A newborn with MSUD typically appears normal at birth. Initial signs and symptoms may include vomiting, irritability, lethargy, poor suck at the end of the first week of life, and a maple syrup odor in the cerumen or urine. If left untreated and a diagnosis is not made immediately, progression can occur including developmental delay, intellectual disability, seizures, coma, and death.^1^, ^7^, ^8^

Screening for MSUD in the Philippines is mainly done by newborn bloodspot screening at the age of 24 h. The diagnosis is confirmed by elevated allo‐isoleucine and elevations of other branched‐chain amino acids. The acute management of MSUD patients in crisis generally involves the removal of accumulated branched‐chain amino acids by peritoneal dialysis or hemodialysis. However in the country, only peritoneal dialysis is being done in tertiary referral centers. Similar to most countries, long‐term interventions in our setting include protein restriction or diet control and monitoring of biochemical marker levels.^4^, ^6^ While liver transplantation is an option in most developed countries, this procedure has not been done in our cohort of patients.

In the country, although early diagnosis through NBS and acute management can be done for patients with MSUD, clinical outcomes remain poor.^4^ Classic MSUD still remains a morbid and potentially fatal condition. Reported mortality rates of patients ranges from 16.7% to as high as 72.2%.^9^, ^11^ This large discrepancy in mortality rates among countries may be due to several factors such as implementation of screening programs, differences in health infrastructure, access to treatment, nutritional support, diet adherence, availability of medical expertise in managing metabolic disorders, follow‐up care and genetic diversity that may also influence severity of presentation. Strict dietary therapy may maintain metabolic parameters within acceptable limits but is challenging to implement, fails to restore appropriate concentration among amino acids, and does not fully prevent cognitive and psychiatric abilities.^10^

**FIGURE 1:**
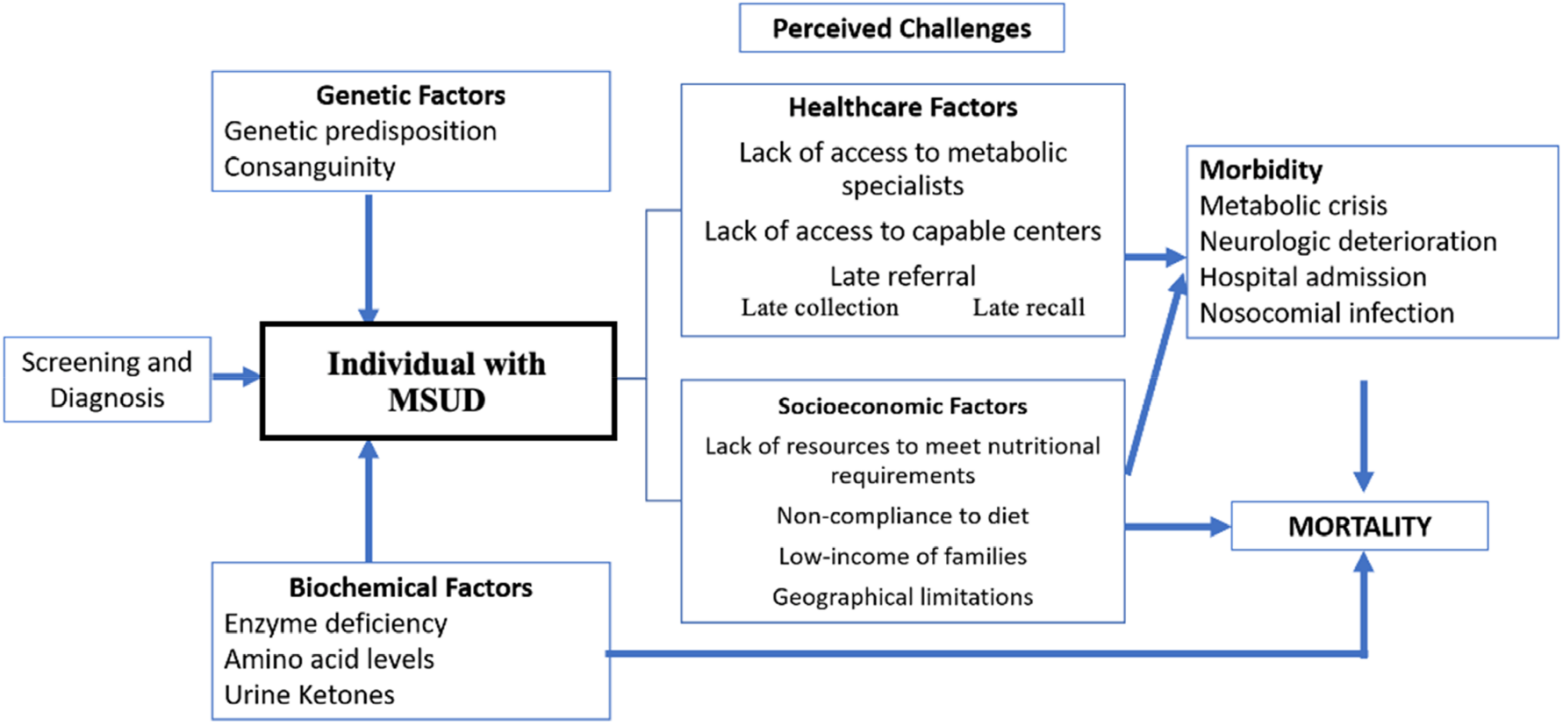
A conceptual framework describing the factors that lead to morbidity and mortality in patients with maple syrup urine disease.

Our literature review included studies that reported the incidence of patients with MSUD across different countries, their risk factors, and their neurodevelopmental outcomes over time (summarized in Table 1, ^4^, ^6^, ^9^, ^10^, ^11^, ^12^, ^13^, ^14^, ^15^). Limited studies ventured into identifying factors associated with certain outcomes. Several socioeconomic, biochemical, and healthcare‐associated factors that potentially relate to important clinical outcomes such as mortality and other morbidities were considered and included in the conceptual framework of this study (Figure 1).

**TABLE 1:** Summary of studies that documented incidence, clinical outcomes, and several factors relating to the outcomes of MSUD patients.

Authors, country (year of publication)	No. of cases/participants with MSUD	Incidence	Variables explored/risk factors (if any)	Clinical outcomes recorded
Quental et al., Portugal (2010)^9^ 1977–2009	36 (32 with classic form and 4 with mild variant form)	Before NBS: 1/113 459 live newborns (95% CI 1/172, 400 to 1/84, 745) After NBS: 1/86 800 live newborns (95% CI 1/3, 937 000 to 1/44 000)	Age at diagnosis confirmation (days of life) Plasma leucine at diagnosis Clinical presentation	Normal neurodevelopment (11/36 cases, 30.5%) Slight, Moderate, severe neurodevelopmental delay (9/36 cases, 25%) Learning difficulties Spastic diplegia/ataxia Episodes of decompensation Death (6/36 cases, 16.7%)
^11^Tu, Wen‐Jun, China (2011) 1996–2011	18 (as mentioned in case reports)	1/200 000 to 1/35 000 infants	—	“Good results when they left hospital” (3/18, 16.7%) Cerebral palsy (1/18, 5.5%) Mild neurologic deterioration (1/18, 5.5%) Died and gave up treatments (13/18 cases, 72.2%)
Lee et al., Philippines (2008)^4^ 1999–2004	47 (26 cases from 1992 to 1998 series, 21 cases from 1999 to 2004 series)	—	Age at onset of symptoms Age at diagnosis Unusual smell Initial diagnosis of sepsis Metabolic control (leucine levels)	Outcomes compared between cases diagnosed from 1992–1998 vs. 1999–2004: Peritoneal dialysis (58% vs. 62%) Mortality rate (27% vs. 24%) Follow‐up rate (74% vs. 87%)
Bouchereau et al., France (2017)^12^	21	—	Long‐term metabolic balance (BCAA levels) Severity of acute metabolic imbalances Leucine blood levels at diagnosis Time to toxin removal procedure	Neurocognitive evaluation: Verbal Intelligence Quotient (IQ) Performance IQ
Morton et al. (2002)^13^	36	—	Neonatal factors, diet control when well, control of catabolism during illness, etc.	Biochemical measurements (plasma leucine levels, rates of decrease, etc.) Rate of hospitalization Developmental outcomes
Hoffman et al. (2006)^14^	24	—	Longitudinal plasma leucine level	IQ score (Intellectual outcome)
Chiong et al. (2016)^15^	26	—	Plasma amino acid levels Urine organic acid levels	Neurologic features: developmental delay/intellectual disability (88%), speech delay (69%), seizures (65%)
De Castro‐Hamoy et al., Philippines (2017)^6^	24	—	Age at diagnosis Metabolic control Ventilatory support Need for IV antibiotics Nosocomial infection Anemia Protein deficiency	No/mild developmental delay Moderate/severe developmental delay Death (8/24, 33%)
Strauss et al., USA (2020)^10^	190 (184 with reliable data, 175 had molecular confirmation)	—	Genotype (*BCKDHA, BCKDHB, DBT*) Classic, intermediate intermittent MSUD Metabolic phenotype Liver transplantation	Survival/Mortality MSUD Biomarkers Cerebral amino acid influx estimates Neuropsychiatric outcomes (early development, FSIQ, affective illness)

At present, there are a number of studies in the Philippines that describe the prevalence of IEMs and few studies that explore the clinical outcomes of patients with MSUD. Specifically, there is very limited literature exploring factors that are associated with poor outcomes of MSUD patients. Thus, this study aims to identify several risk factors and determine the association or causal relationships of these factors with poor outcomes in patients with MSUD who were seen at a tertiary hospital in the Philippines over a 10‐year period.

## 2 MATERIALS AND METHODS

### 2.1 Study design

This is a single‐center, retrospective cohort study involving a review of medical records of all MSUD patients seen at the Philippine General Hospital in a 10‐year period.

### 2.2 Study population and setting

The study population was comprised of charity patients diagnosed with MSUD seen at the Philippine General Hospital (PGH). PGH is the only institution in the country where metabolic geneticists who handle patients with IEMs are presently affiliated.

#### 2.2.1 Inclusion criteria

- All charity patients with MSUD who were seen at the Philippine General Hospital from 2010 to 2019.
- Diagnosed cases of MSUD based on the metabolic registry of the Institute of Human Genetics—National Institute of Health (IHG‐NIH).
- Patients co‐managed outside PGH and were able to follow up at the PGH Genetics Clinic.

#### 2.2.2 Exclusion criteria

- Patients with MSUD seen before 2008 and after 2019.
- Patients not confirmed to have MSUD by confirmatory tests (plasma amino acid, urine organic acid analysis).
- Patients with MSUD co‐managed outside PGH who have died in a different hospital and have never consulted at PGH.

### 2.3 Sample size

According to De Castro‐Hamoy et al.,^6^ there have been more than 120 cases of MSUD diagnosed since 1992–2014 (Metabolic Registry, Institute of Human Genetics, National Institutes of Health, UP Manila). Due to the low incidence of cases, the recruitment of patients was exhaustive wherein all patients with confirmed MSUD seen in PGH from 2010 to 2019 were included in the study and data analysis as long as they fit the inclusion and exclusion criteria. A total enumeration of all patients within the period was done.

### 2.4 Study procedure

Patients with MSUD seen from 2010 to 2019 were identified from the patient databases of the UP PGH Department of Pediatrics and the Division of Clinical and Metabolic Genetics. Records of all patients identified were accessed in the PGH Records Section and the Clinical Genetics Unit at the National Institutes of Health. Necessary permits to be allowed access to the patient records were processed. The medical records were reviewed and demographic, socioeconomic, biochemical, and clinical data as stated in the objectives, if available, were collected and recorded (refer to Table 2 for the operational definitions of these variables). The principal investigator reviewed the patient records and gathered the needed data. Uncertainty in the variables recorded was discussed and decided upon by the principal and co‐investigator.

**TABLE 2:** Demographic profile of MSUD patients seen at Philippine General Hospital from 2010 to 2019.

	No. of patients (%)
Age group (*n* = 75)	
<6 months^a^	23 (31)
6–12 months	4 (5)
>12 months to 2 years	14 (19)
>2 to 5 years	22 (29)
>5 to 15 years	12 (16)
Sex (*n* = 75)	
Male	40 (53)
Female	35 (47)
Income status (*n* = 75)	
Low income	36 (48)
Middle‐ to high‐income	6 (8)
No available data	33 (44)
Educational attainment of primary caregiver (*n* = 75)	
Elementary	4 (5)
Secondary	32 (43)
Vocational	3 (4)
Tertiary	16 (21)
No available data	20 (27)
Geographical location (*n* = 75)	
North Luzon	5 (7)
Central Luzon	24 (32)
South Luzon	17 (23)
Bicol Region	10 (13)
NCR (National Capital Region)^b^	19 (25)

^a^ This age group includes alive patients (*n* = 8) and those who died before 6 months (*n* = 15).

^b^ NCR, located in the major island group Luzon, is the capital region of the Philippines and is the country's political, economic and education center. It is home to over 13 million Filipinos and is composed of 16 cities and 1 municipality.^16^, ^17^

### 2.5 Description of variables and measurements

The table of the operational definitions of independent variables (factors) and dependent variables (outcomes) in the study is seen as Appendix S1.

### 2.6 Outcome measurements

The primary outcome of the study was the documented mortality of MSUD patients seen during the 10‐year period. The patient outcome during the last admission was recorded. The relationship or association between the independent variables and mortality was investigated through statistical analysis. The secondary outcomes included were (a) the presence or absence of neurodevelopmental morbidities such as mild to severe developmental delay and seizures and (b) the length of hospital stay (days).

### 2.7 Data collection and encoding

Source documents included medical records accessed from the PGH Records Section and the Clinical Genetics Unit of IHG‐NIH. The metabolic registry of patients was under the custodianship of the Institute of Human Genetics. The use of data in the registry complied with the Data Privacy Act of 2012. Access to these documents was made with approval of the IHG Director and all the rules that the institute set forth after the approval of the UPMREB. All data gathered from the medical records and registry were kept confidential. Recorded data were anonymized during compilation and analysis to ensure privacy and confidentiality of the patients in the study.

### 2.8 Data analysis

Descriptive statistics was used to summarize the demographic and clinical characteristics of the patients. Frequency and proportion were used for categorical variables, median and inter quartile range for non‐normally distributed continuous variables, and mean and SD for normally distributed continuous variables. Independent Sample *T*‐test, Mann–Whitney *U* test and Fisher's Exact/Chi‐square test was used to determine the difference of mean, rank and frequency, respectively, between patients with and without outcome. The odds ratio and corresponding 95% confidence intervals from binary logistic regression were computed to determine significant predictors for mortality and/or morbidity. All statistical tests were two‐tailed tests. Shapiro–Wilk was used to test the normality of the continuous variables. Missing values were neither replaced nor estimated. Null hypotheses were rejected at 0.05 *α*‐level of significance. STATA 13.1 was used for data analysis.

The patients screened from 2010 to 2014 and those screened from 2015 to 2019 were analyzed in two separate subgroups in terms of the correlation of their leucine levels to the outcomes since the methods of generating leucine levels were different for the respective time periods (Alisei colorimetric testing was used from 2010 to 2014 vs. tandem mass spectrometry [MS/MS] from 2015 to 2019).

## 3 RESULTS

### 3.1 Demographic profile of patients^17^

There were 99 patients diagnosed with MSUD from 2010 to 2019 according to the Institute of Human Genetics Registry. Seventy‐five patient records were available for review at UP‐PGH OPD Records Section and IHG‐NIH. Among the diagnosed MSUD cases, there were 35 (47%) females and 40 (53%) males. Around one‐third of patients (23/75, 31%) were below 6 months old. This group also included 15 patients who died at less than 6 months of age. The majority of patients came from Central Luzon (32%) and the NCR (National Capital Region) (25%). Forty‐eight percent (48%) belonged to low‐income families (Class D, indigents) and 43% had primary caregivers who reached secondary‐level education (Table 2).

### 3.2 Clinical profile of patients

The median age at collection of newborn screening samples among patients was 2 days (interquartile range [IQR] 1–3.5 days) and the median age of symptom onset was 5 days (IQR 4–8 days). Common symptoms include poor feeding, increased sleeping time, seizures, poor activity, and a sweet smell. The median age at diagnosis and age of referral to the PGH metabolic team were both estimated at 7 days (IQR 5–11 days) (Table 3).

**TABLE 3:** Clinical and biochemical profile of MSUD patients seen at PGH from 2010 to 2019.

Clinical parameter					Median (IQR)
Age at collection of newborn screening, days (*n* = 68)					2 (1–3.5)
Age at diagnosis, days (*n* = 61)					7 (5–11)
Age of onset of symptoms, days (*n* = 50)					5 (4–8)
Age of referral to PGH, days (*n* = 61)					7 (6–11)
Time between referral and admission to first hospital^a^, days (*n* = 51)					0 (0–2)
Time between referral and admission in PGH, days (*n* = 10)					3 (1–6)
	**Reference range (μmol/L)**				**Mean (±SD)**
	**2012**		**2016 (revised)**		
	**24–48 h**	**>48 h**	**<7 days**	**≥7 days**	
Initial leucine levels (μmol/L, *n* = 71)	<300	<350	<300	<400	918 (±816)
**Plasma amino acid levels (μmol/L)**	**0–30 days old (μmol/L)**				
Leucine (*n* = 13)	48–160				1404 (±1101)
Valine (*n* = 12)	86–190				470 (±341)
Isoleucine (*n* = 12)	26–91				212 (±232)
Allo‐isoleucine (*n* = 12)	<5				143 (±93)
					**No. of patients (%)**
Urine ketones on admission (*n* = 75)					
Present (trace to +3)					10 (13)
No available data					65 (87)
MSUD type^b^ (*n* = 75)					
Classic					7 (9.3)
Intermittent					1 (1.3)
Mild‐intermittent					3 (4)
No available data					64 (85.3)
Number of crises per year (*n* = 73)					
0					61 (81.3)
1					11 (14.7)
2					1 (1.3)
No available data					2 (2.7)
Peritoneal dialysis (*n* = 75)					
Done					30 (40)
Not done					44 (58.7)
No available data					1 (1.3)
Presence of infection^c^ (*n* = 75)					
With infection on admission					33 (44)
Without infection on admission					13 (17.3)
No available data					29 (38.7)
Co‐morbidities on initial admission (*n* = 47)					
Nosocomial pneumonia/sepsis					17 (36)
Neonatal sepsis					9 (19)
Neonatal pneumonia					5 (11)
UTI					5 (11)
Meconium aspiration					3 (6.4)
Aspiration pneumonia					3 (6.4)
Meningitis					2 (4.2)
Congenital heart disease					1 (2.1)
Ophthalmia neonatorum					1 (2.1)
Omphalitis					1 (2.1)
Metabolic control^d^ (*n* = 75)					
Mean leucine level > 600 μmol/L (poor control)					40 (53.3)
Mean leucine level < 600 μmol/L (good control)					24 (32)
No available data					11 (14.7)
					**Median (IQR)**
Number of times of non‐compliance to diet^e^					1 (0–2)

^a^ Zero number of days indicates that patient was admitted on the same day or within 24 h of referral (reported as median, IQR); 1 day indicates that patient was admitted 1 day after referral and so on. ‘First hospital’ indicates either a local hospital or PGH where patient was first admitted after referral to the metabolic team.

^b^ MSUD classification were mostly based on clinical course. Only one patient classified as intermittent had mutational analysis done.

^c^ Infections include neonatal sepsis (clinical and/or with positive growth on cultures, neonatal pneumonia, nosocomial pneumonia and sepsis, meningitis, ophthalmia neonatorum, omphalitis and urinary tract infection, breakdown of cases seen under *co‐morbidities on admission*).

^d^ Mean leucine levels done on monitoring which may have been taken on either well or sick days. Threshold of 600 μmol/L was based on the guidelines that the Newborn Screening Program has set.

^e^ Dietary non‐compliance was evaluated as non‐adherence to dietary prescription within 80%–120% of prescribed NP and/or caloric intake based on the 24‐h diet recall or report of non‐compliance of parents or caregivers.

The mean initial leucine level among patients was 918 μmol/L (SD ± 816). The plasma leucine levels on confirmatory testing (samples taken on the 3rd to 35th day of life) were higher with mean level of 1404 μmol/L (SD ± 1101). As determined by clinical course, there were seven classic MSUD patients, three mild‐intermittent and one intermittent type on record. Sixty‐one patients (81%) did not have any metabolic crisis in a year, 11 patients had at least one crisis and one patient had two episodes in a year (Table 3).

During the initial admission, 30 patients (40%) underwent peritoneal dialysis. Thirty‐three (44%) patients cumulatively had infections. Seventeen patients had nosocomial pneumonia or sepsis while admitted (Table 3).

Forty patients (53%) had poor metabolic control or mean leucine monitoring levels of >600 μmol/L. The number of times of non‐compliance to patients to dietary prescription was estimated at a median of 1 (IQR 0–2) (Table 3).

### 3.3 Clinical outcomes

**FIGURE 2:**
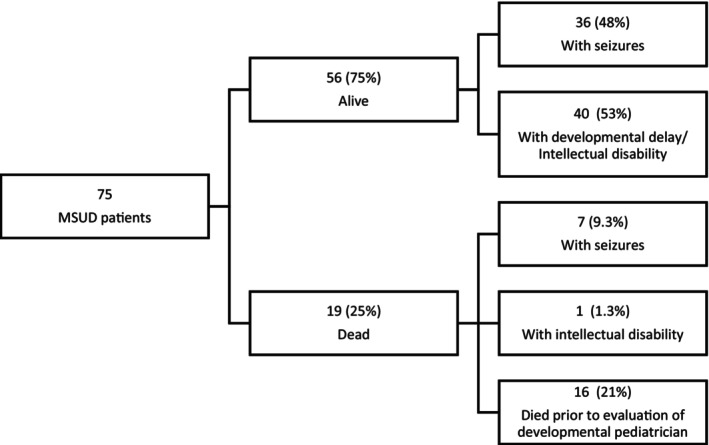
Mortality and neurodevelopmental outcomes of patients seen at PGH from 2010 to 2019. Numbers in parentheses represent proportions in percentage (*n* = 75).

A total of 19 MSUD patients died from 2010 to 2019 with a mortality rate of 25% (19/75). Among these patients, two were documented to be classical type, seven had seizures (9.3%), one had intellectual disability (1.3%) and 16 died prior to formal evaluation by a specialist (21%). Among 56 live patients, 40 (56%) either had a developmental delay or intellectual disability while 36 (48%) had seizures that were either acute symptomatic seizures on admission or seizures with or without electroencephalogram (EEG) findings (Figure 2).

### 3.4 Association of factors and outcomes

#### 3.4.1 Mortality

Patients who were more than 12 months old to 2 years of age were 91% less likely to die compared to patients who were <6 months old (odds ratio [OR] 0.09, 95% CI 0.016–0.499, *p* = 0.006). While patients who were 5–15 years of age were 95% less likely to die compared to patients who were <6 months old with (OR 0.05, 95% CI 0.005–0.446, *p* = 0.008) (Table 4).

**TABLE 4:** Factors associated with mortality and neurodevelopmental morbidity in patients with MSUD.

Parameter	Odds ratio (OR)	95% CI	*p*
Factors associated with mortality			
Age group			
<6 months	(Reference)	—	—
6–12 months	0.18	0.016–2.000	0.162
>12 months to 2 years	0.09	0.016–0.499	0.006
>2 to 5 years	—	—	—
>5 to 15 years	0.05	0.005–0.446	0.008
Age at collection of Newborn Screening (days)	1.29	1.037–1.604	0.022
Number of crises per year	5.43	1.551–19.036	0.008
Presence of infection	0.23	0.058–0.911	0.036
Number of times of non‐compliance to diet	0.29	0.128–0.668	0.003
Factors associated with developmental delay/intellectual disability			
Age group			
<6 months	(Reference)	—	—
6–12 months	7.33	0.36–150.71	0.196
>12 months to 2 years	35.20	3.55–349.15	0.002
>2 to 5 years	198	16.58–2364	<0.001
>5 to 15 years	—	—	—
Male	2.78	1.06–7.26	0.037
Number of times of non‐compliance to diet	2.56	1.48–4.42	0.001
Factors associated with seizure occurrence			
Age group			
<6 months	(Reference)	—	—
6–12 months	0.43	0.04–4.82	0.496
>12 months to 2 years	3.25	0.78–13.48	0.104
>2 to 5 years	3.90	0.96–15.82	0.057
>5 to 15 years	6.50	1.15–36.57	0.034
Nosocomial pneumonia/sepsis	6.96	1.33–36.53	0.022

^a^ Binary logistic regression was used to determine significant predictors for mortality and morbidity (developmental delay and seizures).

For everyday increase in the age at collection of the Newborn Screening, the odds of mortality significantly increase by 29% (OR 1.29, 95% CI 1.037–1.604, *p* = 0.022). For every increment of 1 crisis per year, the odds of mortality significantly increase by 5.4 times (OR 5.43, 95% CI 1.551–19.036, *p* = 0.008) (Table 4). Reasons for the metabolic decompensation include moderate to severe community‐acquired pneumonia, dehydration, and viral infection (with exanthem and pneumonia).

For the patients who had an infection on admission, the odds of mortality were decreased by 77% (OR 0.23, 95% CI 0.058–0.911, *p* = 0.036). Finally, for every increase in the number of times that patients were non‐compliant to diet, the odds of mortality decreased by 71% (OR 0.29, 95% CI 0.128–0.668, *p* = 0.003) (Table 4).

#### 3.4.2 Developmental delay/intellectual disability

In relation to their age groups at the end of 2019, patients who were at the age of above 12 months old to 2 years (OR 35.2, 95% CI 3.55–349.15, *p* = 0.002) and above 2–5 years (OR 198, 95% 16.58–2364, *p* < 0.001) were significantly more likely to have developmental delay compared to those patients who were <6 months old (Table 4).

Male patients were 2.78 times more likely to have developmental delay or intellectual disability compared to female patients (95% CI 1.06–7.26, *p* = 0.037). Finally, in relation to diet compliance, for every increase in the number of times that patients did not comply, the odds of developmental delay/intellectual disability also significantly increased by 2.56 times (95% CI 1.48–4.42, *p* = 0.001) (Table 4).

#### 3.4.3 Seizures

Patients who were between 5 and 15 years of age were 6.5 times more likely to have seizures compared to patients who were <6 months old (95% CI 1.32–36.53, *p* = 0.034). Patients who had nosocomial pneumonia or sepsis during initial admission were around seven times more likely to have seizures (95% CI 1.33–36.53, *p* = 0.022) (Table 4).

### 3.5 Length of stay

The mean length of stay of patients (*n* = 46) who were admitted during their initial crisis was 22 ± 12.8 days.

## 4 DISCUSSION

The newborn screening for maple syrup urine disease in the Philippines was formally initiated in July 2012^6^ and since then, improvements in the program have been employed to be able to provide the utmost care for the patients. Recognition of system changes that address challenges in diagnosis and management of MSUD is vital but the evaluation of clinical outcomes of patients is equally essential to provide information on how patients are handled at the individual level. To date, the last study that described the risk factors and trends in outcomes of diagnosed MSUD cases was the publication of De Castro‐Hamoy et al. in 2017. They described the outcomes of 24 patients from all over the Philippines diagnosed in 2012 to 2014 after the initiation of screening for MSUD.^6^ Using the mentioned study as a basis and noting that MSUD patients grew in number over the past years, this present study determined the association of socioeconomic, healthcare‐related, and clinical factors with mortality and neurodevelopmental morbidity of patients seen in PGH over a period of 10 years.

### 4.1 Socioeconomic and geographical factors (see Table 3)

Among the 75 patients, it was noted that almost half (48%) belong to low‐income families. Since the patients referred to our institution are under the charity service, this trend was expected. While some services such as medical food and doctor fees are free, the cost of other needs such as plasma amino acid monitoring, isoleucine and valine supplements, physical or occupational therapy and transportation for regular consults are largely out‐of‐pocket. Thus, financial constraints among families can consequently affect compliance of patients to the clinician's recommendations and likewise, instill great burden. As reported in a study by Packman et al.^18^ which assessed psychosocial issues in families with MSUD, majority (75%) stated that care for a child with MSUD is a great financial burden especially if there is no or inadequate health insurance.^18^ Similarly, in the absence of a universal health insurance in the country in the past years, the patients' limited finances affect access and compliance to medical management causing adverse outcomes. In this light, we surmised that socioeconomic status may possibly be associated with mortality and neurodevelopmental morbidity. However, it was not seen to be statistically associated with these outcomes.

Based on an unpublished feasibility study assessing possible factors associated with poor outcomes in patients with MSUD, other modifiable variables that can be examined are knowledge of parents/caregivers on the disease and compliance of caregivers to the prescribed diet and follow‐up consults.^19^ Short of having a measure to better assess the knowledge and compliance of caregivers (e.g. quantitative study using questionnaires), we thought of looking initially into the educational status of patients' primary caregivers as these can give us a background on how they would likely comprehend and apply instructions during consults. The results reflect that primary caregivers mostly reached the secondary level of education (43%) while around 21% were able to reach college education. However, there is also no statistical association between this factor and our chosen outcomes. Currently, there are also no other studies relating educational attainment to morbidity and mortality of MSUD patients.

At the time of writing, there are seven Newborn Screening Centers (NSCs) established at the three major island groups in the Philippines—4 NSCs in Luzon, 2 in Visayas, and 1 in Mindanao. Because the country is an archipelago, the receiving and processing of dried blood spot samples may still be affected by the prolonged travel time and limitations in courier services from different provinces in the respective regions catered by the NSCs. In addition, the metabolic specialists in the Philippine General Hospital receive referrals for management of MSUD patients all over Luzon which accounts for 8 of the 17 regions in the country. It has an estimated area of 40 420 square miles, a population of around 62 million, and accounts for 57% of the country's total population.^16^, ^17^ Since the scope of the referral system is quite wide, geographical location was also considered as a variable that can affect outcomes as distant places of origin may contribute to their capability to access metabolic specialists and medical supplies. For the patients diagnosed from 2012 to 2014, inadequate primary medical facilities that can cater to MSUD patients and transferring to a well‐equipped facility were the perceived challenges.^6^ In this cohort, 13 of the 19 patients who died were cumulatively from other regions apart from NCR and most patients transferred to PGH for specialized care. Contrary to the increased number of mortalities outside NCR, there was no association seen between the geographical location of patients and their mortality.

### 4.2 Clinical and biochemical factors (see Table 4)

After birth, the age at collection of newborn screening, diagnosis, recall, referral to a specialist, and the age on admission after referral, all contribute to early and prompt intervention for MSUD. These factors were described in Table 4. Comparing these values with the study of De Castro‐Hamoy et al.,^6^ the median age was earlier in this study for these parameters: age at collection of NBS—2 days (4 days in previous study), and age at referral—7 days (9.5 days in previous). This suggests better execution of the mandate to do the screening after 24 h and that time of referral to the metabolic team somehow improved. However, it should be noted that the data analysis also involved patients who were late‐diagnosed or those who did not undergo newborn screening and were referred to PGH beyond the newborn period. Reasons for referral among these patients include being an older sibling of a patient diagnosed with MSUD through NBS or presenting with developmental delay or intellectual disability.

From the abovementioned factors, the age at collection of newborn screening was found to be associated with mortality. The odds of dying increase by 29% for everyday increase in the age at the collection of the newborn screening (Table 4). Again, this finding emphasizes the importance of early detection of MSUD and justifies the mandate of doing the screening after the first 24 h of life in the Philippines.

It was also seen that patients ages 12 months to 2 years and 5–15 years had significantly lower odds of dying compared to those less than 6 months (Table 4) signifying that fewer children die after infancy and after reaching 5 years of age. In comparison, among 23 patients who were less than 6 months of age, 15 patients (65%) died. This suggests that age might be more of a confounding factor as naturally older patients are less likely to die having surpassed the critical period in infancy. Thus, there is a need to emphasize that more attention must be given especially to those patients less than 6 months old in terms of medical management to avoid mortality in our setting.

Another key factor that was considered to affect patient outcomes was initial leucine levels. The mean leucine levels of patients were 918 μmol/L (SD ± 816, please see reference range in Table 3) which was lower than previously reported by Hamoy et al. (1067 μmol/L ± 529). It must be noted that the mean leucine values that we obtained were generated using two different methods—via Alisei colorimetric testing from 2010 to 2014 and via MS/MS from 2015 to 2019 but separate analysis between these two groups did not show any significant association with our outcomes.

Plasma levels were only available in very few records. In this case, the estimate of branched‐chain amino acid levels might not also be as accurate and should be interpreted with caution in relation to our outcomes. Based on the available data for plasma amino acid levels, these were not seen to be associated with mortality or neurodevelopmental morbidity. This is quite consistent with the results of the case–control study by Chiong et al. in 2016 which correlated plasma amino acid and urine organic acid levels profiles with neurologic features of 26 Filipino MSUD patients. Most of the patients had developmental delay/intellectual disability (85%), speech delay (69%), and seizures (65%). Their amino acid profiles showed significantly low glutamate and alanine as well as high levels of leucine, isoleucine, allo‐isoleucine, phenylalanine, and threonine compared to their matched controls (*p* < 0.05). However, none of these biochemical parameters were shown to significantly correlate with their neurologic manifestations.^15^

Aside from the initial biochemical levels, the classification of MSUD patients into mild‐intermittent, intermediate, or severe types was also a factor of interest as it dictates the manifestations of patients early in the neonatal period.^20^ Classification of MSUD patients seen on record was mainly based on their clinical courses with a majority (7 of 11 patients) presenting as the classical or severe type. Mutation studies are not commonly done in our setting due to the limited financial resources of patients.

Two studies involving genotypic descriptions of MSUD patients were available in the Philippines. A novel deletion in the dihydrolipoyl transacylase (E2) gene was found in 8 of 13 unrelated families, with 5 of them having homozygous mutations signifying a founder mutation in Filipino MSUD patients. Population screening of the deletion revealed that a carrier of the mutations was seen in 1 for every 100 Filipinos.^21^ In 2008, another study reported a deletion in the E2 (*DBT*) gene which facilitated early diagnosis and determined the proper course of treatment in two neonates at risk for MSUD.^22^ The genotypic data would have been useful to determine appropriate management in many instances but was not feasible.

Another variable considered in the prevention of mortality in patients is the number of metabolic decompensations per year as mortality increased by five times for every increment of one crisis annually (Table 4). Most of these patients had community‐acquired pneumonia as the precipitating factor for the crisis and most also belonged to the low socioeconomic status. In literature, illnesses such as respiratory tract infections, gastroenteritis, and cytomegalovirus as causes of hyperleucinosis and encephalopathy are also prevalent even in MSUD patients who already undergone liver transplantation.^23^ For patients who have frequent hospital admission due to hyperleucinosis accompanied by encephalopathy from intercurrent illnesses, non‐related orthopic liver transplantation is ideal.^23^ But as this may be difficult for our patients, more efforts should be made to emphasize preventive measures such as completing *Haemophilus* *influenzae*, pneumococcal, and other vaccinations through the expanded program of immunization, observing proper hygiene to avoid the spread of infection in their homes, and ensuring high caloric intake during intercurrent illnesses to prevent further catabolism and hyperleucinosis.

While patients have increased mortality due to metabolic crisis frequently caused by pneumonia, it was contrastingly seen that patients who had an infection during their initial crisis and admission were less likely to die (OR 0.23, 95% CI 0.058–0.911, *p* = 0.036) (Table 4). The possible reason for this is that patients brought to hospitals for the management of their initial crisis were likewise treated promptly for the intercurrent infection thus avoiding further catabolism and an increase in the leucine levels that can lead to unfavorable complications and death.

With regards to neurologic morbidities, we found out in this study that patients who had nosocomial pneumonia and/or sepsis during initial admission were associated with increased odds of having seizures (95% CI 1.33–36.53, *p* = 0.002). Aside from seizures presenting as a symptom of a possible central nervous system (CNS) infection, severe systemic infections can also present with seizures even though the infections are not present in the CNS. In this case, the causes of seizures include hypoxia and severe metabolic changes like hyponatremia as a result of the severe infection.^24^ This implies that MSUD patients having hospital‐acquired infections due to prolonged hospital stays should also be monitored for seizures and would probably need frequent surveillance from neurologists.

Another factor that may affect mortality in patients is the need for extracorporeal measures for the removal of toxins during initial or subsequent crisis episodes. Forty percent (30/75) of our patients underwent peritoneal dialysis (PD). Since limited facilities in the provinces are capable of doing this procedure, especially for neonates with MSUD, most patients are referred to our institution. Despite a number of patients that underwent peritoneal dialysis, however, it was not seen to be associated with mortality or even neurodevelopmental morbidity. In other countries, PD is not the only option for extracorporeal toxin removal, venovenous hemofiltration and hemodialysis have also been used. These methods are considered in critically ill patients who are comatose and with other associated abnormalities such as refractory acidosis or other electrolyte abnormalities.

A cohort study in 2019 compared continuous venovenous hemofiltration (CVVHDF) versus PD in neonates with inborn errors of metabolism (5 out of 40 patients have MSUD), venovenous hemofiltration was found to be more efficient in eliminating toxic metabolites compared to PD but not in improving survival.^25^ Based on this, in an ideal setting, hemofiltration should probably be considered primarily when it is available more than PD but families should also be apprised regarding catheter‐related complications such as infection and blockage for both PD and CVVHDF.^25^ But in our setting wherein patients are admitted in institutions where resources and expertise for doing extracorporeal toxin removal in neonates is not available, peritoneal dialysis may not always be a priority. Medical management should always be maximized through high‐calorie intake, BCAA‐free feedings, and supplementation with isoleucine and valine.^26^

On the other hand, this study did not find any association between metabolic control (mean leucine levels taken through dried blood spots) and our outcomes. The leucine monitoring levels as a measure of metabolic control should be interpreted with caution in relation to the outcomes since these do not reflect purely leucine levels but are a combination of the leucine, isoleucine, and allo‐isoleucine concentrations when MS/MS is used. It may have also been influenced by supplementation with isoleucine. However, it is known that ideal monitoring for MSUD patients to assess adequate dietary BCAA is through regular plasma BCAA measurement.^27^

Frequent leucine monitoring in MSUD patients is necessary due to dietary restriction in BCAAs and inappropriate intake largely impacts growth, nutritional status, and neurologic status. Contrary to our hypothesis that dietary non‐compliance will be associated with increased mortality, we found out that for every increase in the number of times that patients were non‐compliant to diet, the odds of mortality significantly decreased by 71% (OR 0.29, 95% CI 0.128–0.668, *p* = 0.003) (Table 4). The possible reason for this is that the majority of patients who had frequent records of non‐compliance based on diet review were in the older age groups (>12 months) and these patients were also the ones who were not likely to die. In addition, non‐compliance may also be associated with late‐onset disease as these patients may not need to strictly adhere to protein restriction. The study by MacDonald et al. in 2012 similarly states that dietary adherence deteriorates from age 10 onwards as there is a shift of responsibilities (including feeding) from the caregivers to the patients themselves.^28^ However, while it is not associated with increased mortality, for every increase in the number of times of non‐compliance, patients are 2.56 times more likely to have developmental delay (95% CI 1.48–4.42, *p* = 0.001). As poor dietary compliance is related to poor metabolic control, being non‐adherent to dietary prescription is also likely to cause developmental compromise.

The current sample of MSUD patients included in this study is considered to be representative of the patients in Luzon since PGH receives all metabolic referrals from this region. However, the generalizations in these studies might not be applicable to the subset of MSUD patients seen in Visayas and Mindanao which also have their own referral system and their own set of challenges in the current time.

## 5 LIMITATIONS OF THE STUDY

The review of records only included the patients seen in PGH from 2010 to 2019 and the data included were gathered from available medical records in PGH and the UP‐NIH. There were administrative and system changes in the Newborn Screening Program during the period of interest in this study but these may not be accounted for in this research. The factors included in the review were limited to the socioeconomic, healthcare, biochemical and clinical aspects of care of patients with MSUD. Since this was a retrospective chart review, some information were not found in the patients' medical records. Leucine monitoring levels taken through dried blood spot was interpreted with caution in relation to the outcomes since these did not reflect purely leucine levels but was a combination of the leucine, isoleucine and allo‐isoleucine concentrations. It may have also been influenced by supplementation with isoleucine. The frequency of the leucine monitoring of each patient was also not accounted for. Diet compliance evaluated through the diet recall and report of parents may be subjective and may be influenced by recall bias.

## 6 CONCLUSION AND RECOMMENDATIONS

Several factors were found to be associated with important clinical outcomes in MSUD management. Age at collection of newborn screening and the number of metabolic crises annually were associated with increased mortality. Male sex, dietary compliance, and nosocomial infections were associated with developmental delay and seizures. Although not seen to be statistically significant, the impact of some variables such as the education of the primary caregiver that can dictate compliance to dietary management, MSUD classification, and/or genotyping and monitoring of metabolic control through plasma amino acid levels should not be easily disregarded. The institution of ideal interventions should also be strived upon to be able to correlate factors and outcomes more accurately.

As the review of records also has limitations on how much data will be available for analysis, a prospective study evaluating patients for significant factors such as plasma amino acid levels or metabolic control and dietary compliance is recommended. This will ensure that patient information will be available as desired and that record‐keeping is more organized. The cognitive status and functionality of older patients may also be looked into more closely. Future studies may also explore psychosocial aspects of care and the improvement of the quality of life of our patients.

Finally, we recognize that the current treatment and follow‐up system for MSUD patients needs to be greatly improved in the Philippines. It is our goal to adopt practices in developed countries, with careful consideration, given the geographical, economic, and cultural challenges in our setting.

## AUTHOR CONTRIBUTIONS

Christine Mae S. Avila (primary investigator): Protocol development, conceptualized research design and ensured ethical acceptability, data collection, data analysis and interpretation, manuscript writing and revision, publication and approval of the final manuscript. Mary Ann R. Abacan (co‐investigator and adviser): Reviewed the research protocol and ensured ethical acceptability, provided sufficient oversight and training, data collection, data analysis and interpretation, manuscript writing, assistance in publication and approval of the final manuscript.

## CONFLICT OF INTEREST STATEMENT

The authors declare no conflict of interest.

## ETHICS STATEMENT

The study received ethical approval from the UP‐Manila Research Ethical Board (UPMREB) and followed the National Ethical Guidelines for Health and Health‐Related Research (2017). While there were no direct benefits to patients, the research aims to enhance the understanding of factors affecting outcomes in patients with Maple Syrup Urine Disease (MSUD) and provide recommendations for their care.

The study involved reviewing patient records from a tertiary hospital over ten years, with cases of MSUD confirmed through a registry. The investigators requested for a waiver of informed consent due to minimal risk involved, the non‐sensitive nature of the data, the impracticality of obtaining consent from all families, and the intention to provide relevant information to patients after the study.

Key factors explored included biochemical, healthcare, and sociodemographic elements, with hospital mortality as the primary outcome. To mitigate risks of data privacy breaches, all patient information was anonymized, made confidential, and were securely stored. The study adhered to the Data Privacy Act of 2012 in the Philippines, ensuring transparency and respect for the dignity and autonomy of all individuals involved.

The research promoted the well‐being of persons with disabilities and aimed to involve disability groups in disseminating findings. Funding was provided solely by the principal investigator, who reported no conflicts of interest.

## INFORMED CONSENT

This article does not contain any studies with human or animal subjects performed by any of the authors.

## Supporting information


Appendix S1

## Data Availability

Data archiving is not mandated but data supporting the results reported in the article will be made available upon reasonable request from the investigators of the study.
