# Supplementary material for: Factors associated with poor outcomes in patients with maple syrup urine disease in a tertiary government hospital: A retrospective cohort study
Source: JIMD Rep. 2024 Nov 17;66(1):e12458. doi: 10.1002/jmd2.12458 (PMC11667752; doi:10.1002/jmd2.12458)
Supplement: Supplementary file 1 — Appendix S1 [file JMD2-66-e12458-s001.docx]

**SUPPLEMENTARY MATERIAL 1 (S1)**

**Operational Definition of Variables (factors and outcomes)**

| **Variable** | **Definition** | **Type** |
| --- | --- | --- |
| **Independent Variable (Factors of Interest)** | | |
| Age | Age from birth date to December 2019 (end of study period) for alive patients OR age at time of death (for patients who died) | Continuous |
| Age at NBS collection (days) | Age at the time NBS was collected | Continuous |
| Age of diagnosis of MSUD (days) | Age at diagnosis of MSUD through confirmatory testing as described by the National Institute of Health (NIH) criteria | Continuous |
| Age of occurrence of symptoms (days) | Age at which clinical symptoms of patient started | Continuous |
| Age of referral to a metabolic specialist (days) | Age at which patient was recalled and referred to the metabolic specialists of Philippine General Hospital for management | Continuous |
| Socioeconomic factors | Social and economic factors that characterize the individual within the social structure. Includes data such as, but are not limited to, age, gender, family income, citizenship of patient and parents, level of education of primary caregiver, etc. | Categorical  (nominal) |
| Geographic location | Present home or provincial address of the patient | Categorical  (nominal) |
| MSUD Type/Classification | Refers to the types of MSUD based on clinical course or judgement (classical, intermittent, mild) | Categorical  (nominal) |
| Natural protein (NP) and total caloric intake (TCI) | Refers to the recommended diet in terms of the natural protein (gram per kilo) and total caloric intake of patients (calories) | Continuous |
| Dietary compliance | Refers to the adherence to the dietary prescription as determined by:   - - - 1. Equivalence of NP intake based on the 24-hour diet recall and the NP prescribed or       2. Report of compliance of parents or caregivers | Categorical, dichotomous  (compliant or non-compliant) |
| Biochemical or metabolic markers | Refers biochemical or metabolic substances that are relevant for MSUD patients such as leucine, isoleucine, valine levels, urine ketones, etc. | Continuous/  Categorical |
| Leucine level | The numerical measurement of leucine obtained via dried blood spot or plasma analysis | Continuous |
| Urine ketones | Refers to the measure of the amount of ketones in the urine. | Categorical (ordinal or dichotomous) |
| Presence of infection on admission | Refers to the presence or absence of infection on admission. It will be recorded as either (+) or (-).  This may include, but are not limited to, sepsis, pneumonia, urinary tract infection, etc. | Categorical, dichotomous |
| Metabolic crisis | Refers to an episode of acute clinical decompensation leading to acutely elevated leucine level requiring hospital admission and medical management of the metabolic team. The reason or the cause of crisis shall also be determined (i.e. acute illness or non-compliance to diet) | - |
| Metabolic control | Good metabolic control will be defined as having mean leucine levels of < 600. Good metabolic control will be defined as having mean leucine levels of > 600. | Categorical, dichotomous |
| Dependent Variable (Outcomes) | | |
| Mortality rate  (primary outcome) | Defined as the proportion of patients who died from any given cause at any given time during the included study period | Continuous |
| Neurodevelopmental morbidity (secondary outcome) | Refers to the neurologic and developmental manifestations of patients at the end of the study period. May include but not limited to presence of mild to severe developmental delay, seizures, etc. | Categorical  (nominal) |
| Length of hospital stay (days) | Defined as the duration of hospital admission and calculated by subtracting the day of admission from the day of discharge or mortality. | Continuous |
